# Xicotli Data: a project to retrieve plant-bee interactions from citizen science

**DOI:** 10.3897/BDJ.11.e114688

**Published:** 2023-12-21

**Authors:** Juan M Barrios, Brenda Y Bedolla-García, Paola A González-Vanegas, Andrés Lira-Noriega, Juan C López-Enriquez, Jorge A Mérida-Rivas, Daniel Madrigal-González, Pilar Rodríguez, Matthias Rös, Remy Vandame, Raúl Sierra-Alcocer, Carlos A. Cultid-Medina

**Affiliations:** 1 Comisión Nacional para el Conocimiento y Uso de la Biodiversidad (CONABIO), Mexico City, Mexico Comisión Nacional para el Conocimiento y Uso de la Biodiversidad (CONABIO) Mexico City Mexico; 2 Instituto de Ecología, A. C. Red de Diversidad Biológica del Occidente Mexicano. Centro Regional del Bajío, Pátzcuaro, Mexico Instituto de Ecología, A. C. Red de Diversidad Biológica del Occidente Mexicano. Centro Regional del Bajío Pátzcuaro Mexico; 3 CONAHCYT, Mexico City, Mexico CONAHCYT Mexico City Mexico; 4 El Colegio de la Frontera Sur Unidad San Cristóbal, San Cristóbal de las Casas, Mexico El Colegio de la Frontera Sur Unidad San Cristóbal San Cristóbal de las Casas Mexico; 5 CIIDIR Oaxaca, Instituto Politecnico Nacional, Oaxaca, Mexico CIIDIR Oaxaca, Instituto Politecnico Nacional Oaxaca Mexico; 6 Banorte, Dirección de Analítica Modelaje, Mexico City, Mexico Banorte, Dirección de Analítica Modelaje Mexico City Mexico

**Keywords:** wild bees, host plants, Mexican ecosystems, urban habitats, eco-morphological traits, Apidae, Halictidae, Megachilidae

## Abstract

**Background:**

Xicotli data is the short name given to the dataset generated within the project framework "Integration of Biodiversity Data for the Management and Conservation of Wild Bee-Plant Interactions in Mexico (2021-2023)", as xicotli is the generic word for a bee in Nahuatl. The team comprised eco-informaticians, ecologists and taxonomists of both native bees and flora. The generated dataset contains so far 4,532 curated records of the plants, which are potential hosts of species of three focal families of bees native to Mexico: Apidae, Halictidae and Megachilidae and morphological and ecological data of the plant-bee interactions. This dataset was integrated and mobilised from citizen observations available at naturalista.mx (iNat), which were compiled through the iNaturalist project.

**New information:**

The new information obtained with the Xicotli data project was:

1. Taxonomic information about bee species curated by taxonomists based on the information contained in iNaturalist;
2. Taxonomic identification of the host plants by a botanist from the photos compiled by the Xicotli Data project;
3. Data on the ecomorphological traits of bees and plants based on expert knowledge and literature.

All the data were integrated into the Xicotli Data Project via the creation of new “observation fields". The visibility of the information originally contained in iNaturalist was maximized and can be consulted directly on the iNaturalist platform.

## Introduction

Mexico is a country of bees. Of the nearly 20 thousand species recognised worldwide, approximately two thousand are known in Mexico, around 10% of the global richness (Ascher and Pickering 2020). During the last decade, the information on the interaction networks between native bees and their host plants has increased very little. In five decades for the three most diverse families of bees native to Mexico (Apidae, Halicitidaeand Megachilidae), just over 50 articles have been published focused on this topic (e.g. Arceo-Gómez et al. 2012, González-Vanegas et al. 2021, Wilson 2021).

To face the challenge of knowing the extraordinary diversity of bees in Mexico and effectively assessing the regional and global loss of pollinators, the information obtained from system Creative Commons Attribution License (cc-by) in-situ studies is not enough and it is necessary to take advantage of other sources of information. One of these sources of information is citizen science. At least during the last ten years, citizen-science activities and platforms have become relevant due to their contribution to knowledge about different aspects of biodiversity (Domroese and Johnson 2017, Johnston et al. 2022). An example of these platforms is iNaturalist (Chandler et al. 2017, Marín-Gómez et al. 2022, iNaturalist 2023).

The images people share on iNaturalist featuring a floral visitor and a plant have great potential to become valid information about pollinator-plant interactions. However, for this to occur, additional work is needed. First, the images are focused on the bees, so in iNaturalist, the taxonomic determination is only available for this interaction component. On the other hand, in many images, the plant can be seen with a certain degree of clarity, so identification work carried out by experts would also allow for the taxonomic determination of the other component of the interaction.

The current structure allows the verification and mobilisation of records for one taxon at a time. Precisely, to record information on interactions, it is necessary that, in the same observation, more than one taxon can be recorded, that is, to also be able to include the visited plant.

This article describes the process we followed to transform the images stored in iNaturalist into a dataset containing 4,532 records of taxonomically determined plants associated with floral-visiting bees (Barrios et al. 2023). Additionally, we describe the type of ecological information (ecomorphological traits) integrated into the iNat observations, providing great added value to the dataset and the breadth of geographic, temporal and taxonomic sampling of the biological interactions achieved. The structure we developed is intended to promote the standardised integration of information on pollination interactions and increase the visibility of data on biological interactions between native bees and their host plants.

## General description

### Purpose

To provide open access taxonomic and ecomorphological information on native bee-host plants in Mexico, focusing on flora visited by three highly diverse bee families: Apidae, Halictidae and Megachilidae.

## Project description

### Title

Integration of Biodiversity Data for the Management and Conservation of Wild Bee-Plant Interactions in Mexico (2021-2023)

### Personnel

Juan M. Barrios, Carlos A. Cultid-Medina, Brenda Y. Bedolla-García, Matthias Rös, Rémy Vandame, Pilar Rodríguez, Jorge A. Mérida-Rivas, Paola A. González, Daniel Madrigal, Andrés Lira-Noriega, Raúl Sierra-Alcocer, Juan Carlos López.

### Funding

This project is co-financed by the European Union via GBIF-BID Caribbean National biodiversity data mobilisation grants (BID-CA2020-021-NAC), CONABIO, INECOL, IPN-CIIDIR Oaxaca and ECOSUR.

## Sampling methods

### Sampling description

For the creation of the dataset, five phases were completed: 1) The creation of the iNaturalist project – Xicotli Data: Mexican bees and their flowers; this project compiles all observations (photos with locations within Mexico) that include taxon tags related to three focal families of native bees: Apidae, Halictidae and Megachilidae (i.e. “Honey Bees, Bumble Bees and Allies” OR “Sweat Bees” OR “Mason, Leafcutter, Carder and Resin Bees”). Observations labelled “Western Honey Bee” - *Apismellifera* were excluded. The compilation was unrestricted concerning users, projects, quality grade (i.e. research grade, needs identification), dates and classification; 2) The semi-automatic selection of informative observations (i.e. photos); for this, we use an ensemble of two neural networks, EfficientNet B3 and B7 (Tan and Le 2019), trained to classify the images with flower-no flower. We use the Tensorflow/Keras implementation of such models and CONABIO-ML Vision library to train the model and classify the images (Barrios et al. 2023a). The output of the ensemble model gives the probability of a flower present in the image. We only use those with percentages > 80% (i.e. photos that tended to feature bees and flowers); 3) The creation of “Observation Fields”; this iNaturalist utility allows users to create fields with information that complements the observation data. As taxonomic and location information can only be assigned to one taxon per observation, we use Observation Fields to add data about: i) plant taxonomy and ii) ecomorphological traits (of the plant and the bee); 4) The taxonomic determination of the plants, which was possible for over 37% of the photos with a score > 80%, as the photos showed enough floral structures to carry out a rigorous identification. For this phase, specialised literature like taxonomic descriptions and catalogues was used. Comparisons were made with specimens from two Mexican herbaria: IEB-“Graciela Calderón and Jerzy Rzedowski” and MEXU-UNAM for which a very conservative protocol was applied that guarantees the best possible veracity of the taxonomic determinations; 5) The taxonomic determination of bees; this was carried out simultaneously with the taxonomic determination of plants. Specialist taxonomists hired by the project carried out the taxonomic determination of plants and bees. However, in some cases, help was also received from specialists who follow the Xicotli Data project at iNaturaist. For both bees and their potential host plants, the taxonomic determination was carried out to the lowest possible level without compromising the rigour of the determination (i.e. family, genus, species).

Observation Fields. Seventeen observation fields were created, nine for plants (taxonomy and ecomorphological traits), four for bees (ecomorphological traits) and four for plant-bee interactions. The name of each Observation Field was suffixed with (“XicotliData”), thus distinguishing it from similar fields created by other users or iNaturalist projects. For plants, the final dataset did not include the data for the distribution change (XicotliData) and, in the case of interactions, three of the four Observation Fields were used for the internal work of the research group (i.e. workflow for photo selection). In the following link, the name, definition and value of all Observation Fields can be found: XicotliData project terms dictionary.

## Geographic coverage

### Description

**Figure 1.**
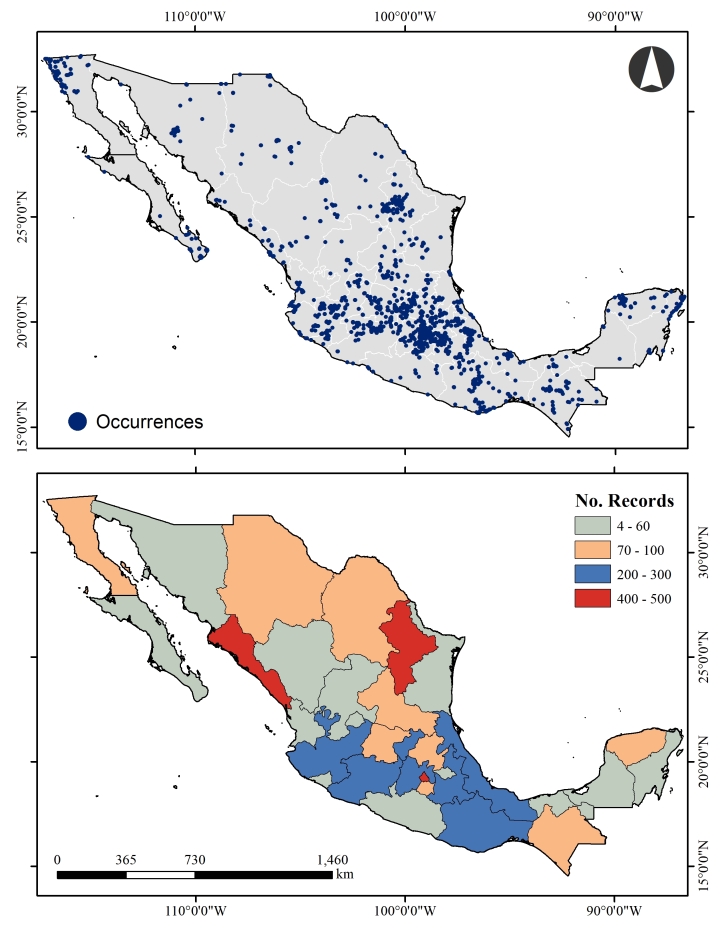
Geographic coverage of the project. **A** distribution of occurrences; **B** the number of records by State.

All considered data are from Mexico. Strong sampling biases become evident when displaying distributional data geographically (Fig. 1).

### Coordinates

14.90556 and 32.64889 Latitude; -118.28925 and -86.71408 Longitude.

## Taxonomic coverage

### Description

It was possible to determine 90 families of host plants. A total of 48% of the observations could be determined only up to the plant genus, 38.1% of the observations were determined up to the species level and 13.9% could be determined only to the family level (Table 1). Of the observations with determinations at the plant species level, 70.3% correspond to interactions with Apidae species, 20.3% with Halictidae and 9.3% with Megachilidae (Table 1). Although the dataset focuses on the taxonomic determination and integration of the ecological information of the host plants, it is important to highlight that, for bees, 44% of the observations were determined at the species and 45.1% at the genus level; only 2.4% of the observations could be made at the level of the bee family (Table 2). Since the dataset has been mobilised through GBIF, the information can be updated as the number of curated observations increases and work continues on the taxonomy and ecological knowledge of native bees.

### Taxa included

**Table:** 

Rank	Scientific Name	
kingdom	Plantae	
phylum	Tracheophyta	
order	Alismatales	
order	Apiales	
order	Arecales	
order	Asparagales	
order	Asterales	
order	Boraginales	
order	Brassicales	
order	Caryophyllales	
order	Commelinales	
order	Cornales	
order	Cucurbitales	
order	Dipsacales	
order	Ericales	
order	Fabales	
order	Gentianales	
order	Geraniales	
order	Lamiales	
order	Liliales	
order	Malpighiales	
order	Malvales	
order	Myrtales	
order	Nymphaeales	
order	Oxalidales	
order	Piperales	
order	Poales	
order	Ranunculales	
order	Rosales	
order	Sapindales	
order	Saxifragales	
order	Solanales	
order	Vitales	
order	Zingiberales	
order	Zygophyllales	

## Traits coverage

### Host plants traits

**Figure 2.**
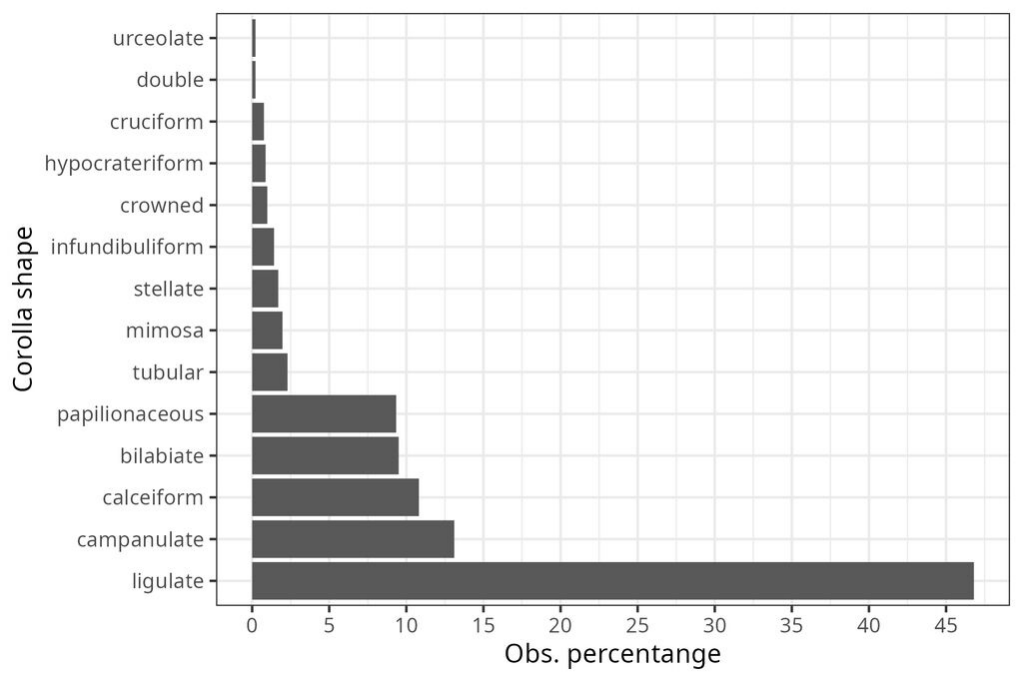
Proportion of the corolla shapes of flowers visited by bees in the registered bee-plant interactions.

**Figure 3.**
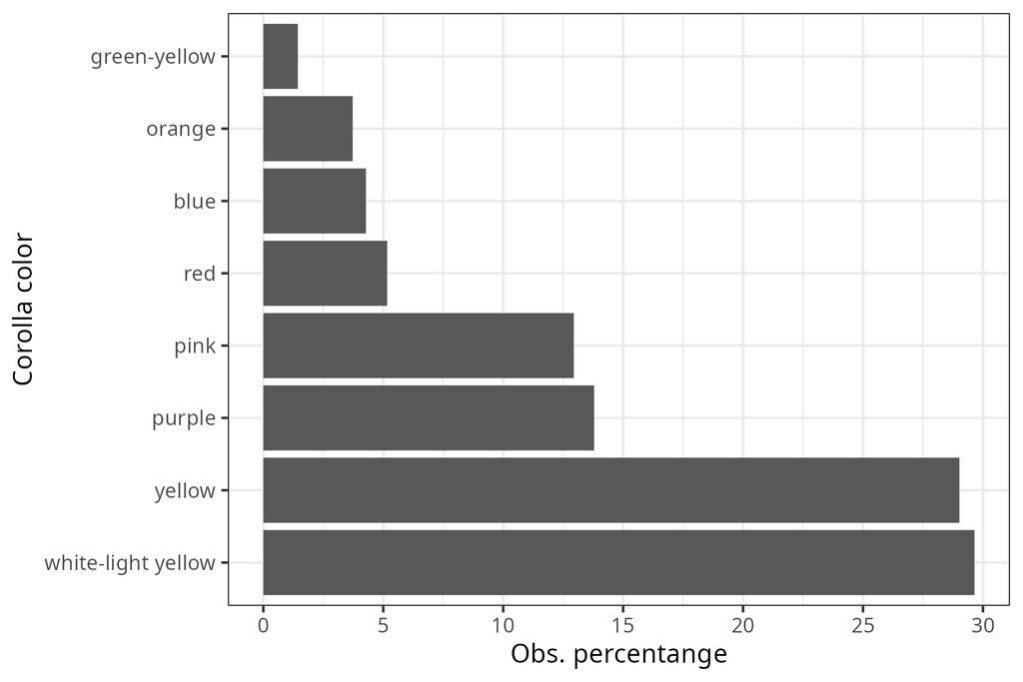
Proportions of the host plant’s records regarding corolla colour.

**Figure 4.**
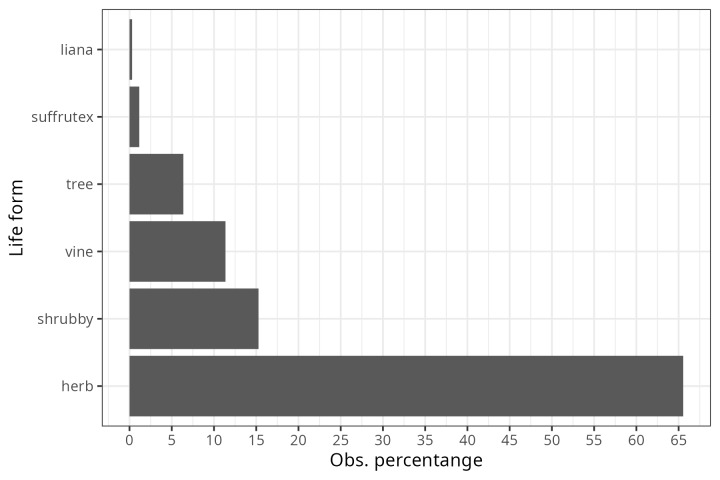
Proportion of plant life forms of the registered bee-plant interactions.

From the inspection of the photographs available for each bee observation, three Observation Fields of ecomorphological traits were completed (as far as possible): i) corolla shape (modified from Moreno 1984), ii) corolla colour and iii) life form. For 1,831 observations, it was possible to assign the shape of the corolla, of which 50% corresponded to native bees visiting flowers with ray-shaped corollas (Fig. 2). A total of 40% of the observations were distributed (in descending order) amongst bell-shaped, cyathiform, bilabiate and papilionate flowers (Fig. 2). The remaining ten corolla forms were distributed amongst the remaining 10% of observations (Fig. 2). The corolla colour was assigned to 2,712 observations and 58.7% of these were grouped into only two colours (Fig. 3): cream-white (29.6%) and yellow (29%). The following most common colours were purple (13.8%) and pink (12.9%) (Fig. 3). The remaining 15% of observations were distributed in the four corolla colours red, blue, orange and green-yellow (in descending order) (Fig. 3). The life form was assigned to 1,303 observations, of which 65.5% correspond to herbaceous plants (Fig. 4), followed by shrubs (15.3%), vines (11.4%) and trees (6.4%) (Fig. 4).

### Native bee-host plants interactions

**Figure 5.**
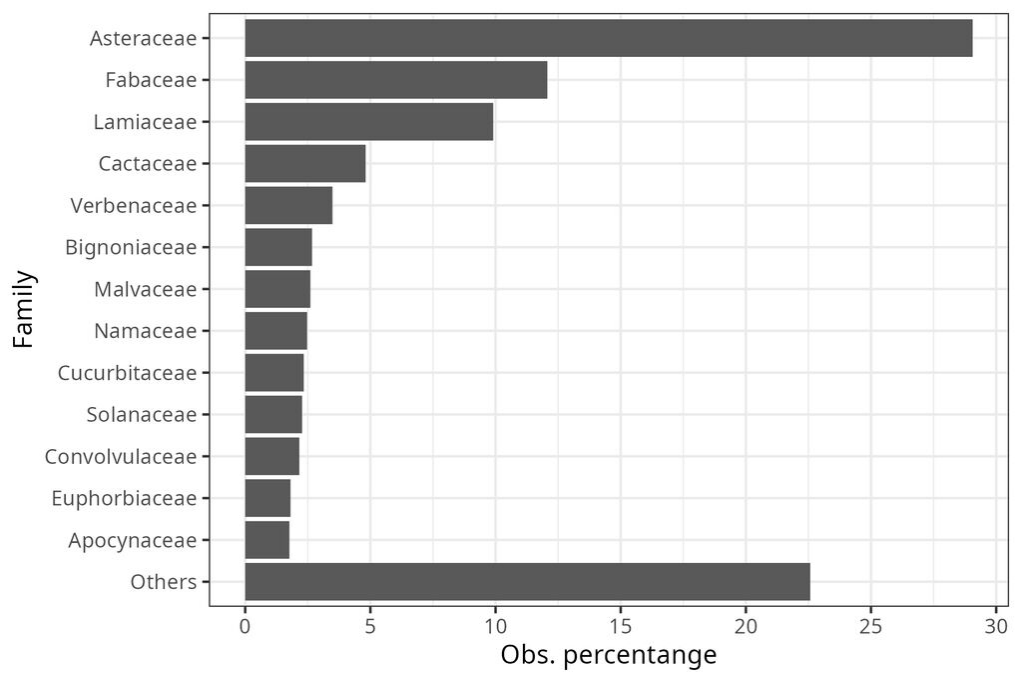
Percentage of bee observations per plant family.

**Figure 6.**
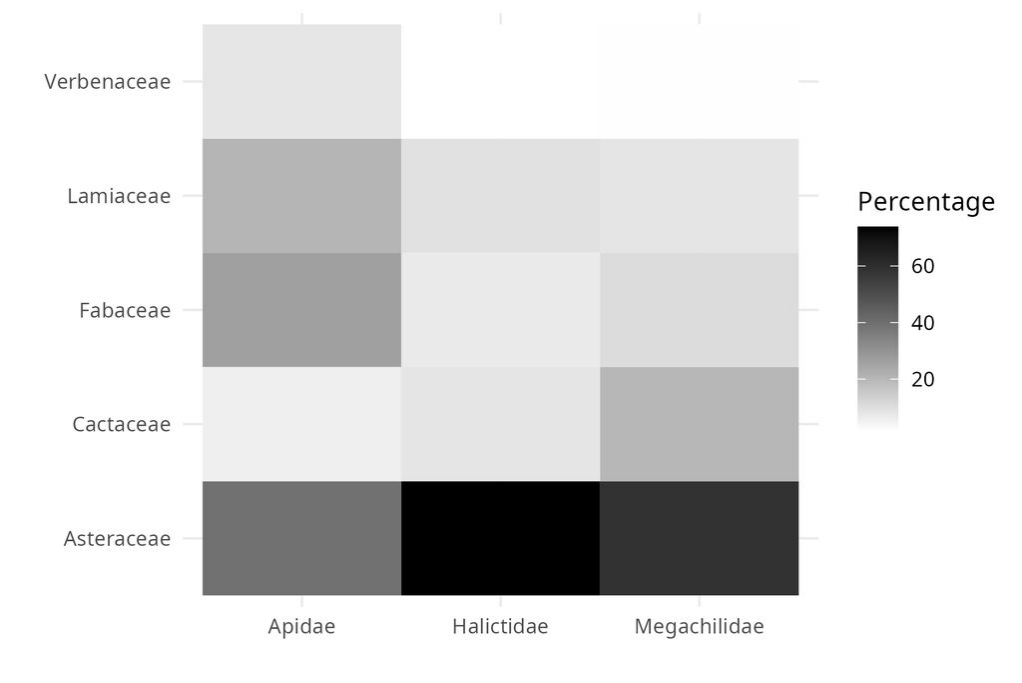
Interaction heat map of interaction plant and bee families.

**Figure 7.**
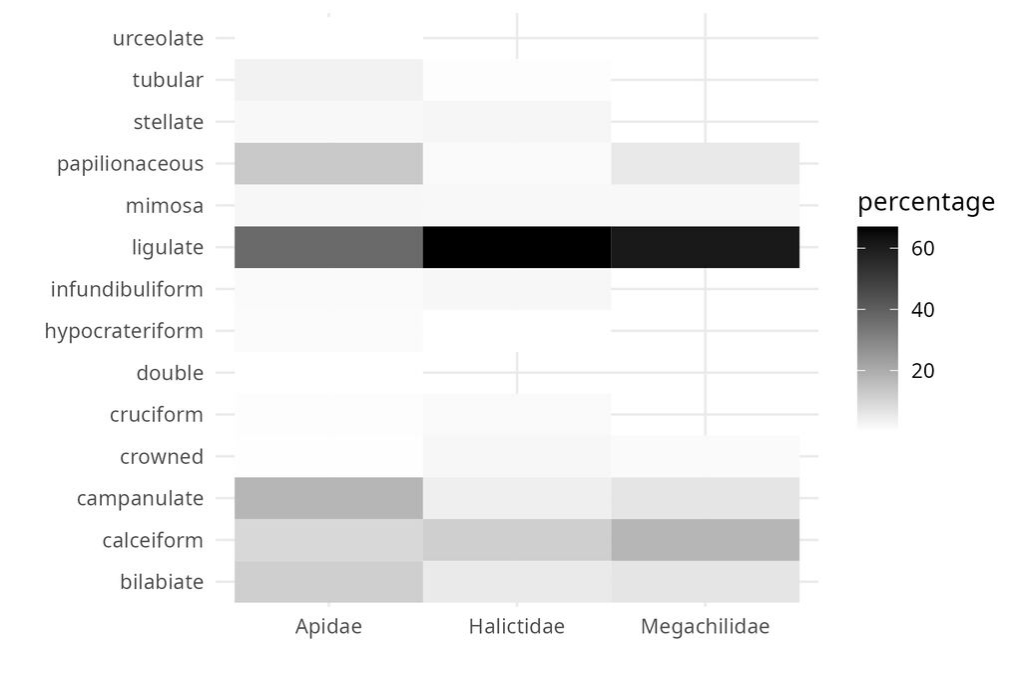
Proportion of the corolla forms of the flowers visited by focal bee family.

**Figure 8.**
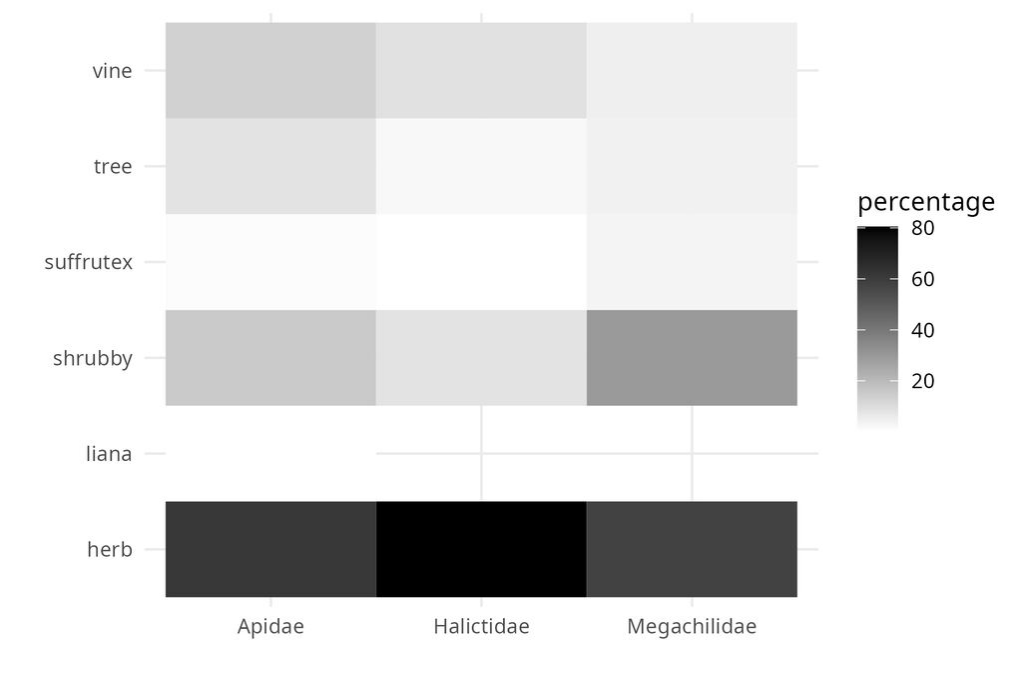
Proportion of life forms visited by the three focal bee families.

Regarding the most common plant families in the evaluated iNaturalist observations (Fig. 5: Asteraceae, Fabaceae, Lamiaceae, Cactaceae and Verbenaceae), the sweat bees (Family Halictidae) were the most frequently detected floral visitors in Asteraceae flowers, with more than 74% of the observations available in the dataset. Leafcutter bees (Megachilidae) and native bees of the Apidae family also concentrated more than 50% of the observations in association with Asteraceae (Fig. 6). However, unlike the other two groups of bees, native Apidae bees were also common in observations that included the other four plant families, most frequently on Fabaceae and Lamiaceae flowers (Fig. 6). It is important to highlight that about 30% of the observations of leafcutter bees were made on cactus flowers. On the other hand, for Verbenaceae, only native Apidae bees have been detected as floral visitors so far (Fig. 6). Consistent with the dominance of Asteraceae in the observations, ray florets concentrate more than 60% of the observations of curated bees from iNaturalist (Fig. 7). Secondly, the calceiform, bell-shaped and bilabiate flowers being second (Fig. 7). Regarding the life forms and the three focal families of native bees, between 70 and 95% of the observations were concentrated on herbaceous plants (Fig. 8). In this way, we hope that the integrated ecological information will be a tool to complement management and conservation strategies for native bee-mediated pollination. Thus, the dataset will be helpful for actions, such as designing pollinator gardens in urban areas.

## Temporal coverage

**Data range:** 2003-8-24 – 2022-8-11.

## Usage licence

### Usage licence

Other

### IP rights notes

Creative Commons Attribution License (CC-BY)

## Data resources

### Data package title

Biological records of potentially host plants of mexican wild bees identified from iNaturalist.

### Resource link

https://www.snib.mx/iptconabio/resource?r=xicotlidata-inat

### Alternative identifiers

https://www.gbif.org/dataset/e1e1e05e-a239-49aa-9e10-94b81cf63b7f

### Number of data sets

1

### Data set 1.

#### Data set name

Occurrence

#### Data format

Darwin Core Archive

#### Character set

UTF-8

#### Download URL

https://www.snib.mx/iptconabio/resource?r=xicotlidata-inat

#### Data format version

2021-07-15

#### Description

Biological records of potential host plants of Mexican wild bees identified from Naturalista (Mexican iNaturalist Node) observations. This is an interinstitutional effort that was carried out for the taxonomic curation and mobilisation of plant observations compiled in the project “Xicotli Data: Native Bees and their Flowers”. Plant observations were obtained from bee photographs available in Naturalista. The project and the resultant dataset of biological records of plants seek to maximise the use of the observations in Naturalista. Thus, it is expected to contribute to the documentation of native plant-bee interactions. This dataset is made up of metadata and three tables: 1) occurrences; 2) measurements or facts table where ecomorphological attributes could be found (see details here) and 3) resource relationship tables, where the type of interaction between bee – plant is recorded (see details here). Taxonomic determination of plants from photographs is a great challenge. However, the plant taxonomists carried out the curation by means of their expert knowledge and by consulting specialised literature (i.e. taxonomic descriptions and catalogues). They also compared the plants from the photograph with specimens of two Mexican herbaria: IEB-“Graciela Calderón and Jerzy Rzedowski" and MEXU-UNAM, applying a very conservative protocol that guarantees the greatest possible veracity of the taxonomic determinations. This dataset was generated within the framework of the project “Integration of biodiversity data of wild bee-plant interactions in Mexico”. The original Naturalista data can be consulted on the following resources: https://doi.org/10.15468/dl.jgd8wd and https://doi.org/10.5281/zenodo.7892227

The dataset is a Darwin Core Archive with Occurrence core and two extensions, ResourceRelationship and MeasurementOrFacts. Therefore, the dataset consists of three tables: occurrences.txt (Occurrence Core), resourcerelationship.txt (Resource Reltionship Extension) and measurementsandfacts.txt (Measurements Or Facts Extension) related by the id field.

**Data set 1.** 

Column label	Column description
id (Occurrence Core)	Darwin Core Archive core id field.
type (Occurrence Core)	The nature or genre of the resource. Value: StillImage.
modified (Occurrence Core)	The most recent date-time on which the resource was changed.
language (Occurrence Core)	A language of the resource. Value: es | en.
licence (Occurrence Core)	A legal document giving official permission to do something with the resource.
references (Occurrence Core)	A related resource that is referenced, cited or otherwise pointed to by the described resource. Value: iNaturalist bee record URL.
institutionCode (Occurrence Core)	The name (or acronym) in use by the institution having custody of the object(s) or information referred to in the record. Value: iNaturalist.
collectionCode (Occurrence Core)	The name, acronym, coden or initialism identifying the collection or dataset from which the record was derived. Value: Observations.
datasetName (Occurrence Core)	The name identifying the dataset from which the record was derived. Value: iNaturalist XicotliData observations.
basisOfRecord (Occurrence Core)	Recommended best practice is to use the standard label of one of the Darwin Core classes. Value: HumanObservation.
occurrenceID (Occurrence Core)	An identifier for the Occurrence (as opposed to a particular digital record of the occurrence).
recordedBy (Occurrence Core)	A list (concatenated and separated) of names of people, groups or organizations responsible for recording the original Occurrence.
eventDate (Occurrence Core)	The data-time of interval during which and Event occurred.
verbatimEventDate (Occurrence Core)	The verbatim original representation of the date and time information for an Event.
country (Occurrence Core)	The name of the country or major administrative unit in which the Location occurs.
countryCode (Occurrence Core)	The standard code for the country in which the Location occurs.
stateProvince (Occurrence Core)	The name of the next smaller administrative region than country (state, province, canton, department, region, etc.) in which the Location occurs.
municipality (Occurrence Core)	The full, unabbreviated name of the next smaller administrative region than county (city, municipality, etc.) in which the Location occurs. Do not use this term for a nearby named place that does not contain the actual location.
locality (Occurrence Core)	The specific description of the place.
decimalLatitude (Occurrence Core)	The geographic latitude (in decimal degrees, using the spatial reference system given in geodeticDatum) of the geographic center of a Location. Positive values are north of the Equator, negative values are south of it. Legal values lie between -90 and 90, inclusive.
decimalLongitude (Occurrence Core)	The geographic longitude (in decimal degrees, using the spatial reference system given in geodeticDatum) of the geographic center of a Location. Positive values are east of the Greenwich Meridian, negative values are west of it. Legal values lie between -180 and 180, inclusive.
geodeticDatum (Occurrence Core)	The ellipsoid, geodetic datum or spatial reference system (SRS), upon which the geographic coordinates given in decimalLatitude and decimalLongitude areas based. Value: EPSG:4326
coordinateUncertaintyInMeters (Occurrence Core)	The horizontal distance (in meters) from the given decimalLatitude and decimalLongitude describing the smallest circle containing the whole of the Location. Leave the value empty if the uncertainty is unknown, cannot be estimated or is not applicable (because there are no coordinates). Zero is not a valid value for this term.
identifiedBy (Occurrence Core)	A list (concatenated and separated) of names of people, groups or organizations who assigned the Taxon to the subject.
identifiedByID (Occurrence Core)	A list (concatenated and separated) of the globally unique identifier for the person, people, groups or organizations responsible for assigning the Taxon to the subject.
nameAccordingToID (Occurrence Core)	An identifier for the source in which the specific taxon concept circumscription is defined or implied. See nameAccordingTo.
scientificName (Occurrence Core)	The full scientific name, with authorship.
nameAccordingTo (Occurrence Core)	The reference to the source in which the specific taxon concept circumscription is defined or implied - traditionally signified by the Latin "sensu" or "sec." (from secundum, meaning "according to").
kingdom (Occurrence Core)	The full scientific name of the kingdom in which the taxon is classified.
phylum (Occurrence Core)	The full scientific name of the phylum or division in which the taxon is classified.
class (Occurrence Core)	The full scientific name of the class in which the taxon is classified.
order (Occurrence Core)	The full scientific name of the order in which the taxon is classified.
family (Occurrence Core)	The full scientific name of the family in which the taxon is classified.
genus (Occurrence Core)	The full scientific name of the genus in which the taxon is classified.
taxonRank (Occurrence Core)	The taxonomic rank of the most specific name in the scientificName.
resourceID (Resource Relationship Extension)	An identifier for the resource that is the subject of the relationship. Value: same as the id.
relationshipOfResourceID (Resource Relationship Extension)	An identifier for the relationship type (predicate) that connects the subject identified by resourcelD to its object identified by relatedResourcelD. Value: http://purl.obolibrary.org/obo/RO_0002623.
relatedResourceID (Resource Relationship Extension)	An identifier for a related resource (the object, rather than the subject of the relationship).
relationshipOfResource (Resource Relationship Extension)	The relationship of the subject (identified by resourcelD) to the object (identified by relatedResourcelD). Value: has flowers "visited by".
measurementType (Measurements Or Facts Extension)	The nature of the measurement, fact, characteristic or assertion. Values: 'corolla colour', 'corolla form', 'life form'.
measurementValue (Measurements Or Facts Extension)	The value of the measurement, fact, characteristic or assertion.
measurementDeterminedBy (Measurements Or Facts Extension)	A list (concatenated and separated) of names of people, groups or organizations who determined the value of the MeasurementOrFact.

## Figures and Tables

**Table 1.** Taxonomic resolution level of plants regarding their visiting focal bee family (4,532 in total).

Bee Family	Plant Id Family	Plant Id Genus	Plant Id Species
Apidae	379	1493	1315
Halictidae	183	462	276
Megachilidae	68	217	137
Unknown	0	2	0

**Table 2.** Taxonomic resolution level for each focal bee family (4,532 in total).

Bee Family	Bee Id Family	Bee Id Subfamily	Bee Id Genus	Bee Id Subgenus	Bee Id Tribe	Bee Id Species	Bee Id Subspecies	Bee Id Complex	Bee Id Missing
Apidae	11	28	1022	229	151	1513	174	59	0
Halictidae	5	56	261	196	231	162	0	10	0
Megachilidae	5	6	301	36	0	73	1	0	0
Unknown	0	0	0	0	0	0	0	0	2

## References

[B9915013] Arceo-Gómez Gerardo, Martínez M. Luisa, Parra-Tabla Víctor, García-Franco José G. (2012). Floral and reproductive biology of the Mexican endemic *Chamaecristachamaecristoides* (Fabaceae). The Journal of the Torrey Botanical Society.

[B9914975] Ascher J, Pickering J Discover Life bee species guide and world checklist (Hymenoptera: Apoidea: Anthophila). http://www.discoverlife.org/mp/20q?guide=Apoidea_species.

[B10929997] Barrios J. M., Lopéz-Enriquez J. C., Rivera-Camacho R., Sierra-Alcocer R. (2023). XD Flower Detection. https://bitbucket.org/conabio_cmd/xd-flower-detection/.

[B9920003] Barrios Juan M, Cultid-Medina Carlos Andres, Mérida Jorge, González-Vanegas Paola Andrea, Bedolla-García Brenda Yudith, González Daniel Madrigal (2023). Biological records of potentially host plants of Mexican wild bees identified from iNaturalist.

[B9915033] Chandler Mark, See Linda, Copas Kyle, Bonde Astrid M. Z., López Bernat Claramunt, Danielsen Finn, Legind Jan Kristoffer, Masinde Siro, Miller-Rushing Abraham J., Newman Greg, Rosemartin Alyssa, Turak Eren (2017). Contribution of citizen science towards international biodiversity monitoring. Biological Conservation.

[B9915050] Domroese Margret C., Johnson Elizabeth A. (2017). Why watch bees? Motivations of citizen science volunteers in the Great Pollinator Project. Biological Conservation.

[B9915059] González-Vanegas Paola A., Rös Matthias, García-Franco José G., Aguirre-Jaimes Armando (2021). Buzz-pollination in a tropical montane cloud forest: compositional similarity and plant-pollinator interactions. Neotropical Entomology.

[B9991133] iNaturalist https://www.inaturalist.org.

[B9915068] Johnston Alison, Matechou Eleni, Dennis Emily B. (2022). Outstanding challenges and future directions for biodiversity monitoring using citizen science data. Methods in Ecology and Evolution.

[B9915077] Marín-Gómez Oscar H., Rodríguez Flores Claudia, Arizmendi María del Coro (2022). Assessing ecological interactions in urban areas using citizen science data: Insights from hummingbird–plant meta-networks in a tropical megacity. Urban Forestry & Urban Greening.

[B10920308] Tan Mingxing, Le Quoc V. (2019). Efficientnet: Rethinking model scaling for convolutional neural networks. In: "International conference on machine learning"..

[B9915086] Wilson Joseph S. (2021). What North American bees are associated with milkweed (*Asclepias*) flowers?. Western North American Naturalist.

